# An overview of CEST MRI for non-MR physicists

**DOI:** 10.1186/s40658-016-0155-2

**Published:** 2016-08-26

**Authors:** B. Wu, G. Warnock, M. Zaiss, C. Lin, M. Chen, Z. Zhou, L. Mu, D. Nanz, R. Tuura, G. Delso

**Affiliations:** 1 GE Healthcare, Waukesha (WI), USA; 2 PMOD Technologies Ltd., Zurich, Switzerland; 3 German Cancer Research Center (DKFZ), Heidelberg, Germany; 4 Peking Hospital, Beijing, China; 5 University of Zurich, Zurich, Switzerland; 6 University Hospital of Zurich, Zurich, Switzerland; 7 Children’s Hospital Zurich, Zurich, Switzerland

## Abstract

The search for novel image contrasts has been a major driving force in the magnetic resonance (MR) research community, in order to gain further information on the body’s physiological and pathological conditions.

Chemical exchange saturation transfer (CEST) is a novel MR technique that enables imaging certain compounds at concentrations that are too low to impact the contrast of standard MR imaging and too low to directly be detected in MRS at typical water imaging resolution. For this to be possible, the target compound must be capable of exchanging protons with the surrounding water molecules. This property can be exploited to cause a continuous buildup of magnetic saturation of water, leading to greatly enhanced sensitivity.

The goal of the present review is to introduce the basic principles of CEST imaging to the general molecular imaging community. Special focus has been given to the comparison of state-of-the-art CEST methods reported in the literature with their positron emission tomography (PET) counterparts.

## Introduction

### MRI and its contrast

Magnetic resonance (MR) imaging is well known for its noninvasiveness and abundance of image contrasts. Conventional MR imaging contrasts are based on the spin relaxation rates of different body tissues under a static magnetic field and various radiofrequency (RF) pulses. Commonly encountered contrasts include T1, T2, proton density (PD), and T2* [1]. The search for novel image contrasts has been the ever-existing driving force in the MR research community, in order to gain further information on the body’s physiological and pathological conditions. Novel image contrasts have been developed by exploiting different aspects: physical or structural properties (diffusion-weighted imaging [2–4], MR elastography [5, 6], etc.); functional properties (perfusion [7, 8], BOLD [9, 10], resting state fMRI [11, 12], etc.); and chemical composition (MR spectroscopy [13, 14], chemical exchange saturation transfer (CEST) [15–22], etc.).

### Why is CEST interesting?

Standard MR imaging relies on the excitation of hydrogen (^1^H) nuclei in water molecules, whose abundance in the human body achieves superior contrast. However, assessing the presence of molecules other than water in body tissues is also of great interest, to probe for chemical compounds and metabolites related to the body’s physiological function and pathological conditions.

A common feature of these chemical substances is that their hydrogen nuclei (as well as other nuclei such as ^13^C) resonate at different frequencies than those of water hydrogen nuclei. For their direct detection using conventional sequences, a multinuclear imaging system (optimized to transmit and receive at frequencies other than that of ^1^H) with possible enrichment could be helpful. However, this is both cost-inefficient and technically difficult.

Proton MR spectroscopy (^1^H MRS) presents an alternative to the multinuclear imaging approach, that can be used on conventional MR systems. A spectral range is imaged, showing the signal amplitudes at different frequencies, which can then be mapped to nuclei in various metabolites resonating at each frequency. In this way, the relative concentrations of different substances may be obtained, in contrast to the information solely on water offered by conventional MR imaging. However, despite the abundant information provided by MRS and its long history (long before MR imaging), it has not quite made its way to daily diagnosis routine. There are several limitations to MRS, due to the low concentration of its targets, compared to water. The primary limitation is the sensitivity: Many substances are difficult to detect at clinical field strengths (3 T and below), both due to limited concentration and spectral overlap of signals from different nuclei, potentially in different molecules (the frequency difference between signals from protons with different chemical shift increases with the strength of the magnetic field). As a result, the acquisition times are long: Many averages are needed for achieving sufficient signal-to-noise ratio (SNR). In addition, MRS has limited spatial coverage/resolution: Most of MRS is single-voxel based, with voxel sizes (in humans) around 20 mm^3^.

Chemical exchange saturation transfer (CEST) practically overcomes some of the concentration limitations of MRS: By exploiting a chemical property of certain metabolites, these can be detected with increased sensitivity—to two orders of magnitude higher or more—by a continuous process of re-saturation and exchange (to be discussed in the coming sections). Furthermore, CEST is based on imaging sequences and can therefore benefit from well-known fast acquisition strategies, as well as improved spatial resolution.

## Review

### Principles of CEST imaging

#### RF saturation and exchange

The principle of CEST is well described by its full name: chemical exchange saturation transfer.

In MR, saturation is a temporary state in which tissue shows no net magnetization. This can be exploited to achieve image contrast, by exciting the sample in a way that only certain tissue types become saturated, showing reduced signal or even entirely disappearing from the image. For example, some fat suppression techniques use saturation at the fat frequency (i.e., the resonance frequency of methylene protons in tri-glyceride molecules) to remove the fat signal from the subsequent imaging.

In CEST, magnetization is transferred from other molecules to water molecules, so that the saturation effect (i.e., signal reduction) that was originally on the targeted species can instead be observed on water. The requirement for this transfer to take place is that the chemical species must have in its structure a ^1^H proton that is exchangeable with those of water.

Hence, the principle of CEST imaging is simple: Given a chemical species of interest, capable of exchanging its ^1^H protons with those of water, a radiofrequency pulse is applied at (one of) its resonant frequency(ies) in order to reach a saturation state. This magnetic saturation will spontaneously be transferred to water over time, via chemical exchange of the excited metabolite protons with non-excited water protons. The subsequent decrease in water signal, which can be conveniently detected by standard MR imaging sequences, will provide an indirect measure for the concentration of the species of interest.

If CEST was limited to what has been discussed above, it would be of very little practical utility: As the chemical species is usually present in very limited quantities compared to water (${10}^{-5}\sim {10}^{-6}$), no noticeable signal change would be observed if a single transfer took place. The core feature of CEST is the continuous transfer of excited ^1^H protons, leading to a buildup of saturation in water. Indeed, when exchanging a saturated proton with water, that proton will be replaced with an unsaturated ^1^H proton from water, which can in turn be saturated for another transfer. If this exchange takes places 100 times, then the detectability of the chemical substance (through the reduction of water signal) is amplified by a factor of 100 compared to other methods.

**Fig. 1:**
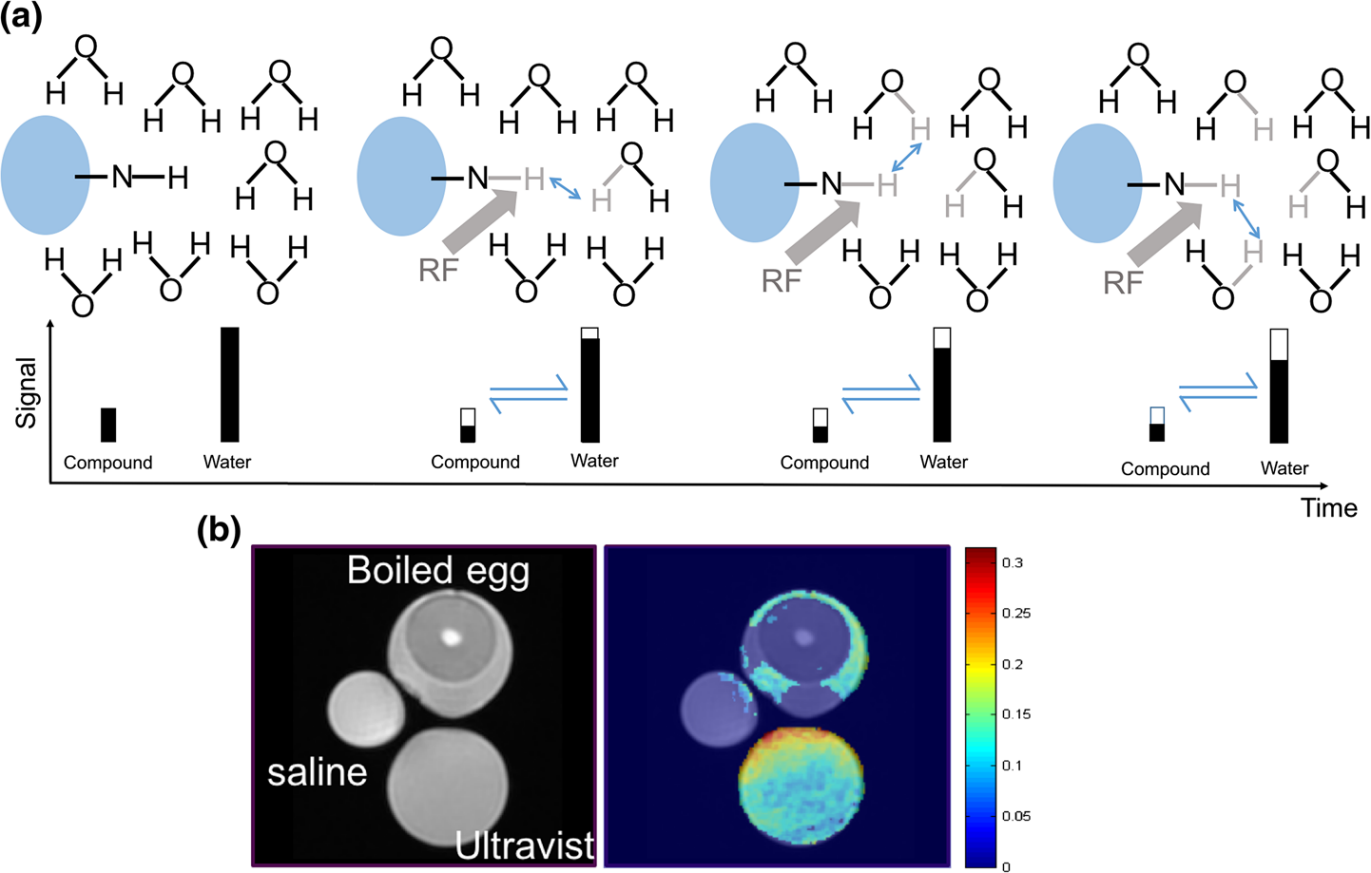
**a** Diagram illustrating the process of CEST: in a solute, the small quantity of chemical substance containing an amine group (-NH) is saturated by a RF, which initially reduces the signal of the substance (shown as the *hollow bar*); then, the saturated hydrogen proton is transferred to water in return for an unsaturated hydrogen; this process continues that leads to amplified water signal reduction (assumes that the saturation level on the chemical substance itself remains unchanged). This process will continue subject to the T1 relaxation and back exchange. **b** Comparison between conventional T2-weighted image and CEST at 4.2 ppm: only Ultravist (Iopromide solution) and egg white yielded CEST contrast

Figure 1a is used to illustrate the concept of the CEST exchange. This exchange will take place at a rate that is dependent on chemical substance concentration, temperature, and pH level and will continue until a steady state is reached (or until the end of the RF saturation of course). The difference between conventional MR image and CEST contrast is shown in Fig. 1b: due to the similar T2 relaxation rates, T2-weighted images show limited contrast between saline water, boiled egg, and Ultravist (Iopromide solution, which is known to contain amide groups that resonate at 4.2 and 5.6 ppm w.r.t. water), whereas the CEST contrast for Ultravist and egg white at 4.2 pm is quite different from the other objects.

For successful CEST contrast to be generated, the exchange process needs to be in the slow to intermediate regime on the NMR time scale [23, 24]. This requirement may be roughly written down as *k*_ex_ < ΔCS, where the exchange rate *k*_ex_ between the chemical substance and water needs to be smaller than the chemical shift $\Delta \mathrm{C}\mathrm{S}$ (i.e., the resonance frequency difference between the compound and water, in radians/sec), in order to be able to distinguish between the two groups. For example, chemical shifts around 3 ppm and exchange rates around 5500 s^−1^ have been reported for brain glutamate and shifts in the 0.5–1.5 ppm range with exchange rates over 10^3^ s^−1^ for muscle Glycogen [19].

#### What can be imaged in CEST?

In principle, CEST is applicable to any solute that contains a chemical substance with an exchangeable ^1^H and abundant water. However, since our primary interest of CEST is its clinical relevance, most CEST applications focus on those metabolites that can be found in the human body or chemical substances that can be externally administrated as a contrast agent. Numerous types of compounds have been investigated for their suitability in clinical CEST imaging, and mainly, they may be classified in two fashions: by their chemical shifts (diamagnetic or paramagnetic) or by the exchange type involved, such as proton exchange, molecular exchange, and compartmental exchange [15, 21]. We adopt the first type of classification for clarity: paramagnetic CEST (paraCEST) agents involve metallic ion and as a result are generally far away from water (as compared to diamagnetic CEST agents), whereas diamagnetic CEST (diaCEST) agents do not involve metallic ions and are usually within 6 ppm from water. ParaCEST agents are usually exogenous and need to be administrated whereas many diaCEST agents are endogenous. Not only do paraCEST agents often require invasive injection, they are also associated with potential metal toxicity. Therefore, one attractive feature of CEST lies in the use of endogenous diaCEST, where a contrast is generated without the need for injections.

#### What is measured in CEST?

As introduced above, CEST allows the presence of chemical species to be indirectly detected, via the reduction of the water signal. However, we have no knowledge of how many times the proton transfer has taken place, which would be required to work out the actual quantity of the targeted compound. Therefore, when it comes to quantification, we are limited to the relative measure provided by measuring the level of water signal reduction caused by the saturation transfer, compared to the corresponding unsaturated image.

This simple concept is complicated with other competing effects, i.e., saturation transfer is not the only process that alters the water signal level, there are other effects that take place during the application of saturation RF. Hence, for reliable CEST quantification, the effects of competing processes need to be eliminated. The main two competing effects are magnetization transfer contrast (MTC) and direct water saturation (DS).

Magnetization transfer is itself a contrast often utilized for neurological imaging. MTC has a quite similar nature to CEST, where saturated protons are exchanged with non-saturated protons in water. However, there are two distinctive differences between MTC and CEST: firstly, MTC involves semi-solid macromolecules with a very short T2* time and hence a very broad spectrum; secondly, the MTC effect is not spectrally specific and is more heavily affected by the bandwidth of the saturation pulse than its spectral position.

The direct water saturation effect can be easily understood: since the spectral profile of the saturation RF is never perfect (i.e., side lobes are present), RF irradiation centered on metabolite resonance frequencies inevitably also affects, to a certain degree, the water-proton magnetization.

In order to eliminate the effects of MTC and DS, instead of comparing the signal reduction caused when saturating a specific spectral location (+τ ppm w.r.t. water) to that without saturation, the CEST effect is assessed by comparing with the water signal reduction when saturating the opposite spectral location (−τ ppm). This assumes that the MTC and DS effects are symmetrical about the water frequency. In this way, the most commonly utilized CEST metric (MTR_asym_) at +τ ppm is derived as:

$$ {\mathrm{MTR}}_{\mathrm{asym}}\;\left(+\tau \right)=\frac{S_{-\tau }-{S}_{+\tau }}{S_0} $$

Where *S*_+τ_ and *S*_−τ_ are the measured signal with RF saturation at +τ and −τ, respectively, and *S*_0_ is the signal measurement without RF saturation.

**Fig. 2:**
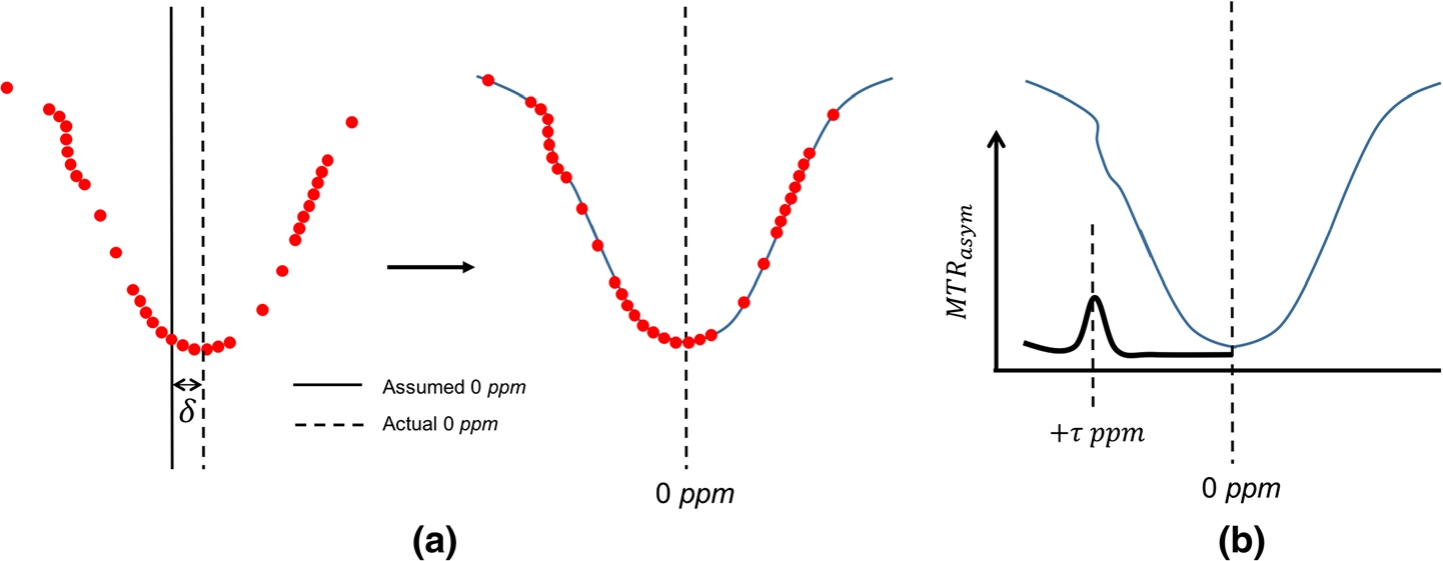
**a** B0 inhomogeneity causes the entire z-spectrum to be shifted, causing a mismatch between the assumed and actual 0 ppm position. Dense sampling (*red dots*) around the expected water frequency allows the frequency shift α to be derived (often performed with a low power short-duration RF), which may be used to shift back the z-spectrum; **b** the MTR_asym_ curve may be derived after the B0 field correction, and CEST effects are observed around τ ppm as indicated by the peak in MTR_asym_

As shown above, to derive the quantification metric MTR_asym_ for an exchanging chemical compound at *τ* ppm, in theory, only CEST measurements at two opposite spectral locations plus a reference scan (at 0 ppm) are needed. However, in practice, this assumption is disrupted by the presence of magnetic field (B0) inhomogeneity. In addition to the slowly varying field inhomogeneity intrinsic to the MR hardware, a more concerning field inhomogeneity is caused by the specific susceptibility of patient tissue. The consequence of field inhomogeneity is a position-dependent shift of the entire spectrum, i.e., *S*_+τ_ and *S*_−τ_ are no longer at their assumed positions, making the calculation of MTR_asym_ meaningless. This is illustrated in Fig. 2a, where the entire spectrum is shifted by an unknown amount $\delta$.

To overcome this issue, knowledge of the frequency shift per voxel is needed so that all the measurements can be shifted back to their designated spectral positions (to compensate $\alpha$). This can be achieved by densely sampling the spectral region around water (i.e., using RF saturation pulses at different frequency offsets around the expected frequency of water) and finding the minimal point after interpolation (i.e., where we would be saturating the real frequency of water). This is illustrated in Fig. 2. In addition, regions around +τ and −τ also need to be densely sampled, so that the proper *M*_+τ_ and *M*_−τ_ may be recovered from the neighboring points. With sparse sampling of the rest of the curve and proper interpolation, we obtain what is denominated the z-spectrum. MTR_asym_ for any point on the curve may then be calculated, as illustrated in Fig. 2b. It is also possible to use external B0 field maps for this correction [25–27].

#### CEST implementation

In general, MR pulse sequences can be thought to be composed of two parts: magnetization preparation and data acquisition. The former part determines the type of image contrast, whereas the latter part plays a more important role in the resulting image quality and scan time. Due to the unique contrast formation in CEST, both parts require special attention for its clinical application.

The most commonly used type of RF for CEST saturation is a continuous wave irradiation, i.e., a long rectangular RF pulse. This is the most widely used RF pulse to date for its simplicity. An additional advantage of continuous wave is the easy optimization if the prior knowledge of the exchange rate ${k}_{\mathrm{ex}}$ is known, as only the length and height of the RF need to be considered. The shortcomings of continuous wave are twofold: The lengths of most RF pulses in conventional MR imaging are in the order of tens of milliseconds (ms), whereas a RF length in the order of seconds is needed for CEST. This may be limited by the hardware; Another consequence of ultra-long RF pulses is the increased power delivered to the patient, measured by the specific absorption rate (SAR), which may pose a safety issue, especially at high field strength.

A more practical alternative is the use of pulsed saturation, where a train of short (short relative to a continuous wave) RF pulses is used. In this way, the burden on the hardware, as well as the SAR level, may be effectively reduced. In pulsed saturation, more sophisticated RF shapes have been attempted such as Fermi and Gaussian. In addition to the pulse shape, pulsed saturation brings additional factors to take into account for CEST contrast optimization: individual pulse length, inter-pulse gap, number of pulses, etc. For example, it has been reported that the inter-pulse gap may be utilized to improve the specificity of CEST agents [28, 29]. However in practice, the optimization of the saturation RF pattern is often performed experimentally, not only due to the high number of factors but also because the exchange rate in vivo cannot be exactly known [23, 30].

In addition to direct RF saturation, other types of CEST preparations have also been proposed such as frequency-labeled exchange transfer (FLEX) [31–33], saturation with frequency alternating RF irradiation (SAFARI) [34, 35], or indirectly via spin-lock [36–38], which bear potential advantages.

The basic requirements for CEST data acquisition are the same as most other imaging techniques: high SNR and little image distortion within the shortest scan time. However, two needs of CEST imaging place stringent constraints on the scan time: a large number of spectral offsets are needed to adequately sample the z-spectrum (>30) and the long saturation time used (in the order of seconds) which lengthens the necessary repetition time (TR). The former requires multiple instances of image acquisition, whereas the latter limits the minimum time for each acquisition. Under such constraints, both the image spatial resolution and spatial coverage are limited.

**Fig. 3:**
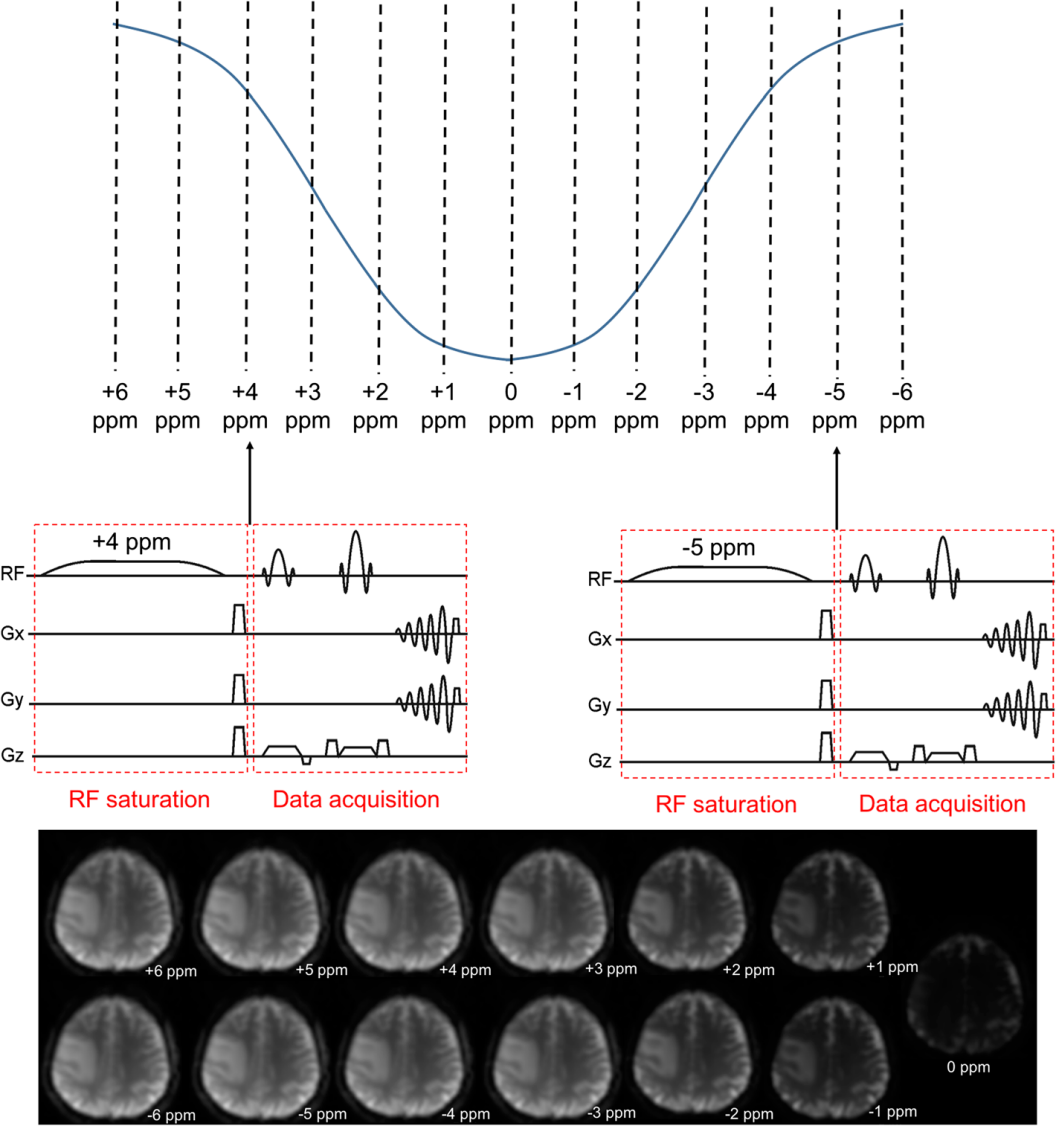
Illustration of the CEST acquisition: saturation RF is played at different spectral positions on the z-spectrum, followed by (usually) single-shot data acquisition. The total scan time is the product of the number of spectral points acquired and the time needed for each image. The relative variation of signal intensity with different spectral offsets can be clearly seen

To speed up the acquisition, 2D single-shot techniques are usually used for CEST, such as single-shot echo planar imaging (EPI) and single-shot fast spin echo (FSE). This means that, for example, if a TR of 3 s is used and 40 spectral points are needed, the acquisition of a single slice will need 2 min, excluding calibration scans. Multi-slice acquisition (for improved spatial coverage) or multi-shot acquisition (for improved spatial resolution) would require multiple times the scan time above. Figure 3 shows a typical CEST acquisition scheme with a sampling step of 1 ppm using a single-shot spin echo spiral acquisition.

These prolonged data acquisition times might be acceptable for motionless body regions such as the brain, pelvis, or extremities, but voxel-based quantification of CEST requires the alignment of all the images acquired at different spectral offsets, which limits CEST imaging of moving organs.

Development of 3D CEST acquisition methods has also been reported using carefully designed data sampling techniques such as 3D-GRASE [39] and 3D-SPIRAL [40].

### Applications of diaCEST

Known endogenous diaCEST agents are typically involved with exchangeable groups of –NH, –NH_2_, and –OH, whose chemical shifts are ~3.5, ~1.8–3.0, and ~0.5–1.5 ppm, respectively [19]. In CEST, some propose a convention to name the type of imaging by the CEST agent involved. Hence, CEST imaging with each of the functional groups mentioned above would be named as amide CEST, amine CEST, and hydroxyl CEST. In the same fashion, CEST imaging using resonances of a particular agent such as glutamate may be referred to as Glu-CEST, etc. In the past years, different endogenous agents and their potential applications have been extensively studied and reported.

#### CEST of amide protons

Amide CEST is the most widely used CEST imaging to date, and it features the most stable and sensitive detection compared to other diaCEST imaging in vivo at 3 T. Amide CEST is also popularly known as amide proton transfer (APT). The major known contributors to APT are the proteins and peptides of the tissue [41, 42]. The main applications reported for APT are the detection of cancer and ischemic stroke.

In tumor regions, the concentration of proteins is elevated compared to surrounding tissues, and subsequently, the increased intracellular exchanges lead to an increased APT level. APT has been demonstrated for tumor grading [43–46], differentiation of tumor from edema [15], and separation of tumor progression from radiation necrosis [42]. Apart from brain tumors, APT cancer detection has also been applied in the breast [47, 48] and prostate [49, 50]. The extension of APT to other body regions, although promising, is complicated by motion issues.

APT imaging of ischemic stroke relies on the variation of pH level: reduced pH in the ischemic region leads to lowered APT exchange rate, and as a result, a decrease in CEST is observed. The advantage of APT imaging of ischemic stroke is detection in the acute stage due to its high sensitivity to pH changes [51, 52].

**Fig. 4:**
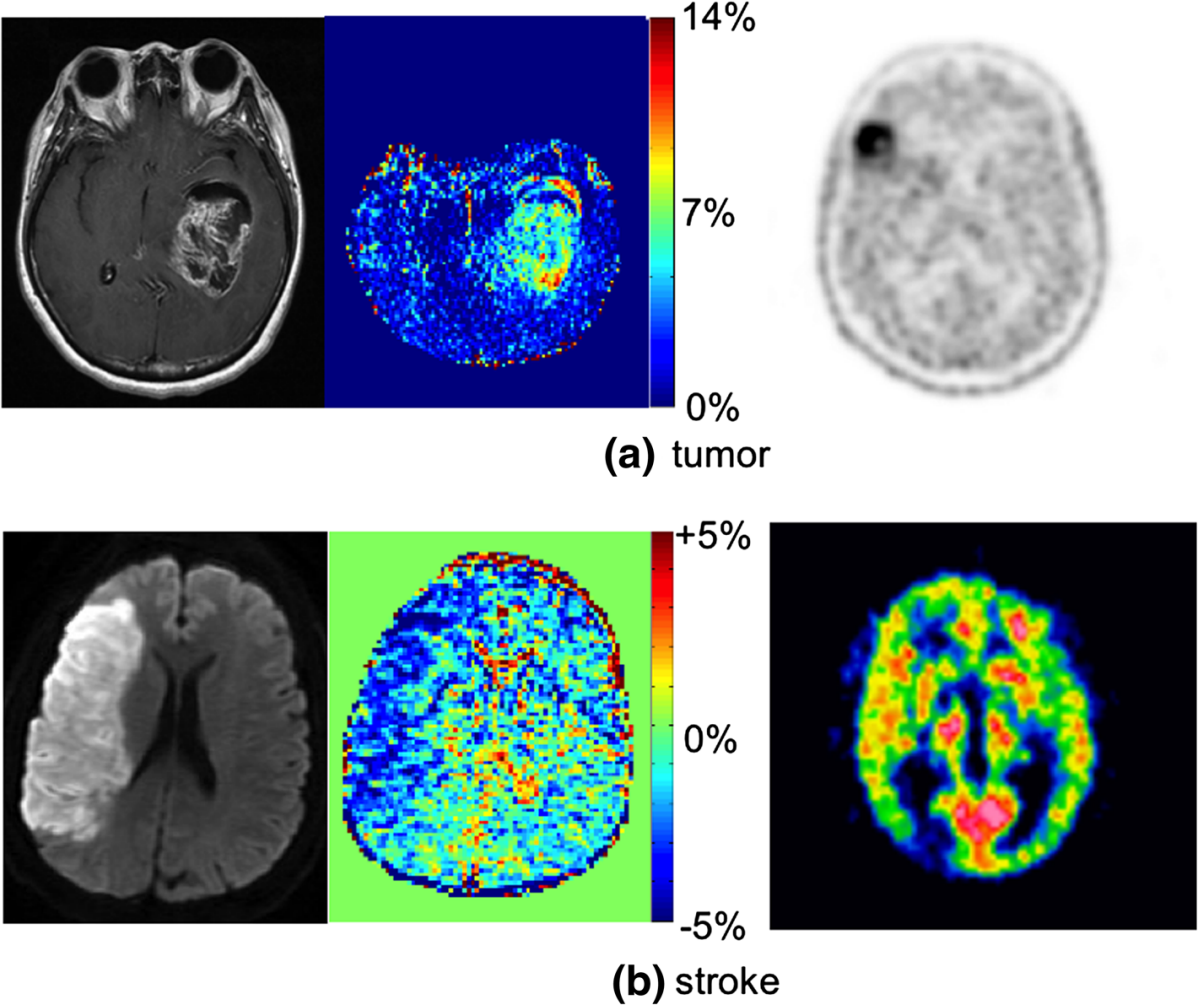
APT (*left*) and PET (*right*) images of **a** tumor and **b** ischemic stroke (MR and PET images are in each case from different patients). In tumors, an increased level of APT is observed, whereas decreased APT is seen in stroke. The PET tracers used were [^18^F]FET and [^15^O]H_2_O, respectively. Altered level of standard uptake levels are observed in the PET images in these cases. The PET images are shown exemplarily; therefore, we did not include any scale bars or quantification information

The APT contrast is hence developed differently in tumor and stroke: in tumor, the exchangeable proton content increases and the change of intracellular pH level is negligible, whereas in ischemic stroke, the exchanging proton content stays relatively constant and the pH level decreases. As a result, elevated and decreased levels of APT are observed in each case, respectively, as illustrated in Fig. 4.

#### CEST of amine protons

Amine groups can be found in amino acids as well as peptides. Two important endogenous metabolites for CEST imaging using amine groups are glutamate and creatine.

**Fig. 5:**
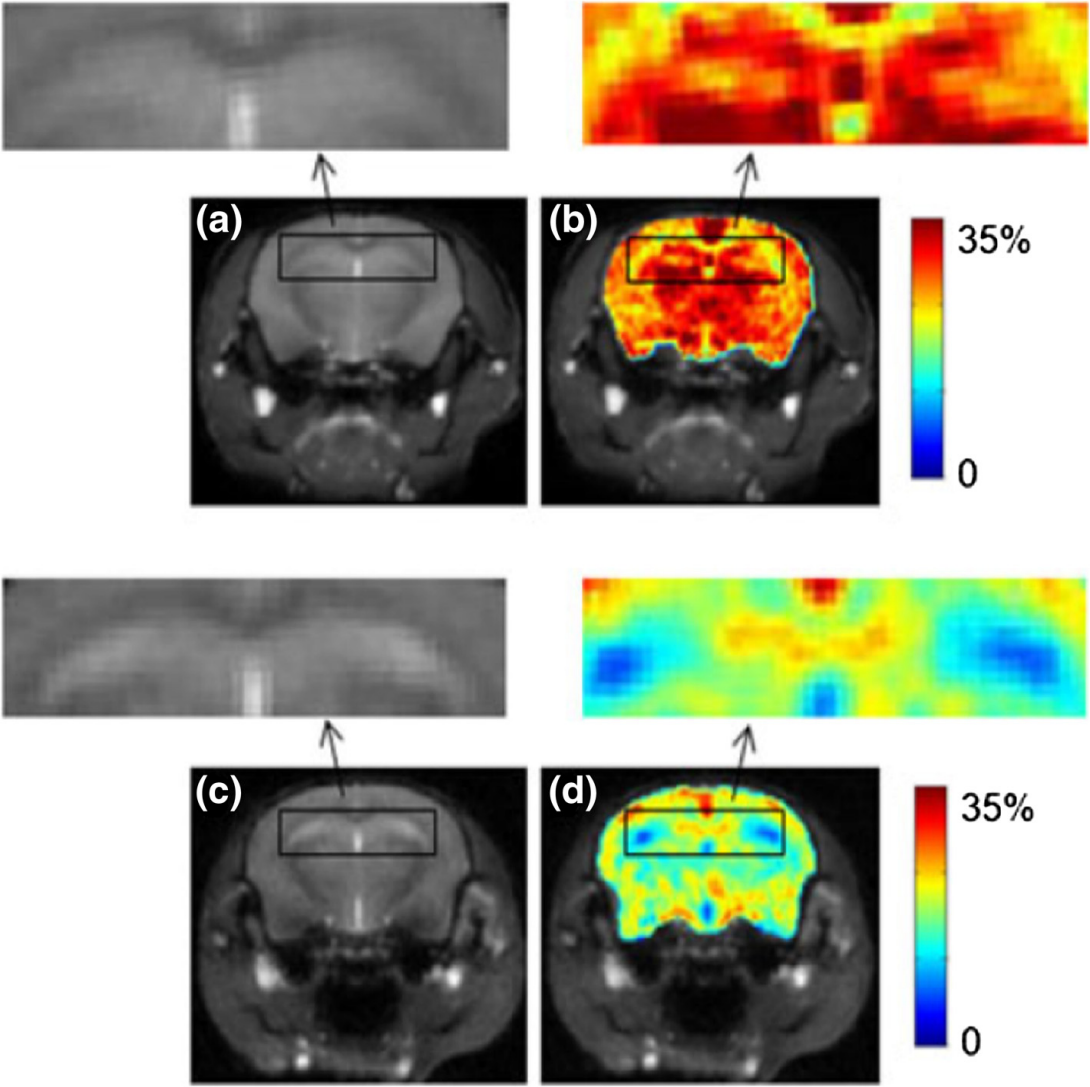
Comparison of anatomical images (**a**, **c**) and Glu-CEST (**b**, **d**) maps in wild-type control (*top*) and Alzheimer’s disease (*bottom*) model mice. Significantly decreased Glu-CEST levels were observed in the hippocampus of Alzheimer’s disease model mice, indicating the potential value of Glu-CEST as a biomarker for AD. (Reproduced from [55] with permission)

Glutamate is an essential neurotransmitter responsible for excitatory transmission in the central nervous system [53]. In vivo mapping of glutamate could provide valuable information for neurological studies and neurodegenerative diseases. Glutamate CEST has recently been demonstrated feasible, and its spatial distribution was verified using PET maps of the metabotropic glutamate receptor subtype 5 [54]. Considerably, lowered Glu-CEST levels have been observed in Alzheimer’s disease (AD) models [55]. The nonuniform changes among different cerebral regions may lead to a better understanding of the involvement of these regions at different stages of AD, which is difficult with conventional MR imaging. Other potential neuro-Glu-CEST applications include middle cerebral artery occlusion (MCAO) [53] and stroke [56]. In vivo mapping of Glu in the spinal cord has also been demonstrated, with potential application as a biomarker for spinal cord-related neurological disorders and spinal cord injuries [57]. Similar to APT, Glu-CEST is also sensitive to pH level variation and may be a viable indicator for pH changes. Figure 5 shows an example of Glu-CEST imaging.

Creatine is the substrate/product of the enzyme creatine kinase (CK), which catalyzes the reversible conversion of creatine (Cr) into phosphocreatine (PCr) [58]. Phosphocreatine (PCr) is consumed in the rapid regeneration of ATP during muscle contraction, which consequently leads to an increase of the Cr concentration. Cr can be used as a biomarker for the muscle energy metabolism under both normal and pathological conditions. Conventionally, in vivo detection of Cr was only feasible via MR spectroscopy [59]. High-resolution Cr-CEST in skeletal muscle (axial section of the calf) showing increased creatine levels immediately after exercise and decrease to normal level during a recovery period has been reported [21, 60]. The ability to map muscular energy metabolism may be useful in the study of myocardial disorders [61].

#### CEST of hydroxyl protons

The hydroxyl (-OH) group resides closest to water among all the diaCEST agents and features a comparatively high exchange rate. Several metabolites with exchangeable hydroxyl protons have been exploited for CEST, such as glycosaminoglycan (Gag), myo-inositol (MI), and glucose.

**Fig. 6:**
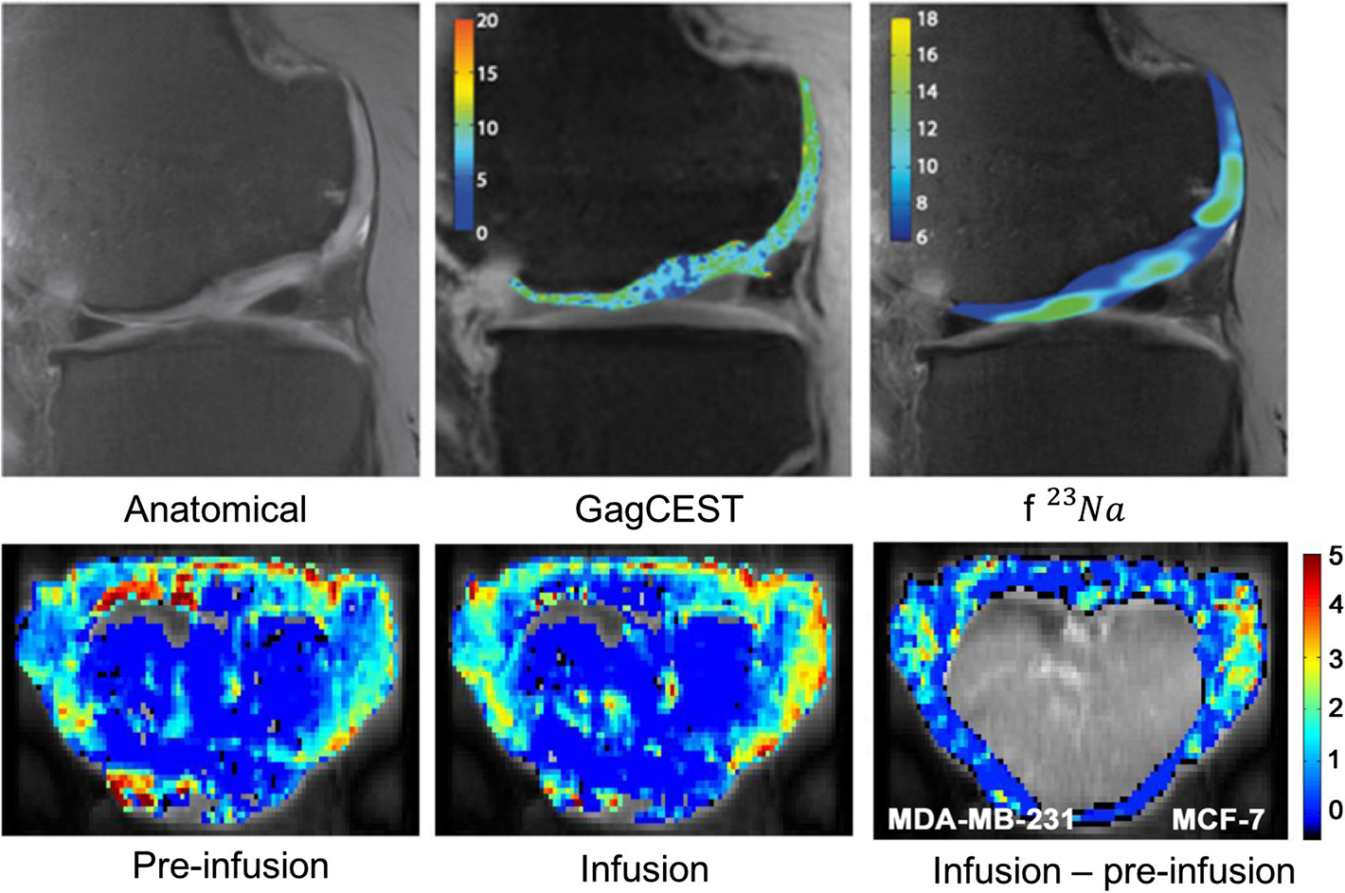
(*top*) Gag-CEST showed a high correlation with the Gag distribution map obtained using ^23^Na MR images in joint cartilage (reproduced from [64] with permission); (*bottom*) d-glucose is infused in mice inoculated with breast tumors, and the difference between Gluco-CEST pre- and post-infusion images shows increased glucose levels in the tumors (reproduced from [67] with permission)

Glycosaminoglycan is an important constituent of cartilage. Its loss and abnormal distribution could be used as an indicator for cartilage damage and degeneration such as that in osteoarthritis [62, 63]. Conventional imaging of Gag using either contrast-enhanced or T1-rho MR has limited specificity [62]. It has been demonstrated that the estimate of Gag distribution obtained using Gag-CEST at 7 T exhibits an excellent correlation with that obtained using MR-based sodium imaging [64], which is considered to be the gold standard for in vivo Gag imaging. Figure 6 shows an example of Gag-CEST imaging.

Glucose is an ubiquitous energy source in the human body. Its uptake and metabolism are sensitive biomarkers for many pathologies, such as stroke, cancer, and various forms of psychiatric/neurodegenerative diseases [65, 66]. Tumors often have an abnormally high reliance on the consumption of glucose, and this is widely utilized in ^18^F-FDG PET imaging for tumor detection and staging. Due to the small spectral separation between glucose and water hydrogen resonances and its limited intrinsic quantity, direct mapping of the intrinsic glucose distribution in vivo is very challenging at clinical field strengths. However, exogenous glucose, such as in the form of d-glucose, may be used as a non-pharmacological contrast agent to facilitate Gluco-CEST [67].

MICEST targets Myo-Inositol, synthetized from glucose-6-phosphate and proposed as a marker of glial cells. It has a nonuniform distribution in the brain and has been shown to increase in the early stages of AD [68]. MICEST has been reported to show considerably elevated levels of Myo-Inositol in models of AD [69] and has potential as a biomarker for other types of neurological disorder.

### Additional practical aspects to consider

As briefly summarized, a number of endogenous CEST agents present exciting potential applications. If we allow the consideration of exogenous agents, which can be chemically developed, a large number of agents are at our disposal. However, as in any other technical development, the translation of laboratory investigation to clinical use requires the consideration of several practical aspects.

#### Is ultra-high field needed for CEST?

In addition to increased image SNR and CEST effects, there is a fundamental benefit in using higher magnetic fields: the chemical shift increases in proportion to magnetic field strength. As discussed previously, for successful CEST, the exchange rate must be smaller than the chemical shift. This impedes the feasibility of detecting several diaCEST agents that reside close to water at 3 T, such as several substances consisting of the amine and hydroxyl groups [70]. To date, CEST imaging of these intrinsic metabolites have only been reported at 7 T and above. Higher field strength also helps to improve the detection specificity when multiple CEST agents are present. For instance, glutamate and amide are only 0.5 ppm apart—which translates to only 64 Hz at 3 T—and both metabolites feature a broad spectral profile [16]. Also amide, amine, and nuclear Overhauser enhancement (NOE) peaks can be easier separated from each other and from the direct water saturation [71]. Another benefit of higher fields is the general longer T1 relaxation time of water. This allows accumulating more saturation in the water pool and thus increases the detectable CEST effect.

The general downsides of higher field strength are also more pronounced for CEST imaging. The susceptibility-related voxel-wise magnetic field shift is proportionally increased with field strength and hence places a more stringent requirement on the B0 correction. RF homogeneity deteriorates severely at 7 T and, if not properly calibrated, a nonuniform saturation profile across the field-of-view may result [27, 72]. The SAR level also becomes a hazardous issue at ultra-high field. Due to the uncommonly long RF lengths needed by CEST, careful design of the RF saturation strategy is needed. Overall, at present, the benefits of 7 T (likely to become a clinically approved field strength in the near future) for diaCEST outweigh the shortcomings [17] and high field strengths are recommended, subject to hardware availability as well as regulations.

#### Quantification model

Given the scale of the CEST effects generally encountered (<5 % at 3 T), an appropriate quantification model that eliminates other compounding effects is desirable. The asymmetrical MTR parameter is the simplest to compute and indeed the most widely used in practice. However, a key assumption underlying the asymmetrical MTR is that of MTC effects, in contrast to CEST effects, being symmetrical about water. Although true for most paraCEST agents whose resonance frequencies are far away from that of water, the MTC effects for frequency ranges in close proximity to water (within 5 ppm) have been reported to be asymmetrical. In a recent study, Scheidegger et al. [35] showed that, with certain types of RF preparation, the observed APT in tumors at 3.5 ppm, at 3 T, could be contributed by asymmetrical MTC effects. One effective method to eliminate the effects of asymmetric MTC is to compare the CEST effects between target tissue and control tissue, as the asymmetric MTC effects may still be comparable. Another factor that must be considered is the nuclear Overhauser enhancement (NOE) effect that occurs within −1.0~−4.0 ppm of water. NOE is a type of cross-relaxation pathway where spin polarization exchange takes place. NOE effects have larger impacts for protons that resonate at frequencies close to water. It has been shown that NOE effects can be mitigated using higher RF amplitude (>2.0 uT) [73, 74] or more complicated models taking into account different exchange pathways. It should be noted that although names like APT, gluCEST, and gagCEST are being used, what MTRasym reflects is CEST effect weighting rather than direct quantification. Additionally, as the water T1 has direct influence on the CEST effect, CEST effects are sometimes normalized by T1 (e.g., when quantification of concentration is the goal); however, depending on the pathology, this can decrease or increase the obtained tissue contrast [56, 71, 75], so this kind of normalization is not always desirable for clinical reading. A systematic review of CEST quantification models is given in [76].

#### Measurement reproducibility

A number of instrumental and physiological factors influence the level of CEST effects. The instrumental factors, including magnetic field strength, RF magnitude, RF design, and B0 and B1 homogeneity, are common to most MR imaging. The physiological factors, including tissue relaxation rates, agent concentration, pH value, and temperature, require specific attention for CEST measurement. In the case of phantom measurements, buffer concentration also has a large effect on the exchange rate and should be accounted for. The sensitivity to environmental factors (pH, temperature, etc.) in fact expands the potential applications of CEST. A good example is the use of APT in the detection of stroke, where pH becomes the parameter of interest. However, this feature inevitably makes reproducibility more challenging.

Another challenge for reproducible CEST measurement in practice is the completeness of the chemical exchange process. Ideally, measurement should be made after the two-way exchange process has reached a steady state. However, most in vivo CEST experiments have been made in the transient state, due to the impractically long RF saturation period needed to reach steady state (>3 s at 3 T).

Due to the factors listed above, CEST longitudinal and multicenter reproducibility may still be a hurdling issue in practice, although encouraging results have been reported in [77, 78].

#### Exchange transfer preparation

Chemical transfer via RF saturation is a classic and commonly practiced method for chemical exchange, however, is not the only way. Another often used approach, sometimes referred as on-resonance saturation, is chemical exchange spin lock (CESL), which employs a spin lock module similar to that used in T1-rho imaging [36–38]. Similar to CEST, CESL can also be performed off-resonant providing an attractive feature that is the freedom to use higher RF power while avoiding increased levels of direct water saturation. This feature is particularly beneficial for imaging agents with smaller chemical shifts, as reviewed systematically in [34, 36].

In contrast to saturation, frequency-labeled exchange transfer (FLEX) [31–33] uses a series of Label Transfer Modules to selectively label the exchangeable protons that could be subsequently transferred to water. A distinctive feature of FLEX, compared to the saturation-based approach, is the fact that the water signal is modulated instead being reduced. Line fitting is used in FLEX analysis and allows the separation of components with different T2*, this is advantageous as short T2* components, attributed to MT effects, may be eliminated [31].

Other approaches have also been investigated such as dual band saturation [79, 80] that has intrinsic insensitivity to asymmetric MT effects. Detailed discussions on these approaches are beyond the scope of this review.

### CEST and nuclear medicine

The recent developments in CEST imaging have caused equal measures of excitement and confusion within both the Radiology and Nuclear Medicine communities. Undoubtedly, CEST methods will enable MR to make further headway into the molecular imaging field (i.e., the visualization and follow-up of biological function at the molecular level, and in particular cellular metabolism). However, there seems to be a great deal of misunderstanding concerning the development status of these techniques and the timeline for their integration into clinical practice.

In particular, Gluco-CEST has been presented as a viable alternative to FDG PET [65, 81]. Quoting Rivlin et al. “Thus 2-DG/FDG CEST MRI can replace PET/CT or PET/MRI for cancer research in laboratory animals, but also has the potential to be used in the clinic for the detection of tumors and metastases, distinguishing between malignant and benign tumors and monitoring tumor response to therapy as well as tumors metabolism noninvasively by using MRI, without the need for radio-labeled isotopes.”

Certainly, the need for short-lived radioactive tracers for PET imaging carries a considerable financial burden, as well as requiring infrastructure (e.g., cyclotron, radiation shielding) and distribution logistics. Furthermore, it entails the delivery to the patient of a significant dose of ionizing radiation. All of these drawbacks might indeed be overcome, in the future, by a CEST method requiring only a few grams of dextrose as contrast.

On the other hand, there are still serious obstacles in order for CEST to get anywhere near its more successful PET alternatives (e.g., the excessive acquisition time and need for a 7-T system). In the first reported use of gluco-CEST in the clinic, 25 g of dextrose was infused intravenously over approximately 1 min [82]. As this resulted in a hyperglycemic state (blood glucose 189–427 mg/dL), the technique in its current form is contraindicated in diabetic subjects (beyond standard MR contraindications such as metal implants and pacemakers). In contrast, FDG PET requires microgram levels of injected tracer due to the exquisite sensitivity of PET. This order of magnitude difference in concentration between PET tracers and MR contrast agents will likely remain true for most CEST applications. Beyond contraindications and adverse reactions, the use of pharmacological doses of biological substrates creates a risk of altering the state of the biological system studied.

The typical radiation dose for an 18 F-labeled PET tracer study (including only low-dose CT) is approximately 5 mSv, which is considered equivalent to 1–2 years of life in a major city and is below the yearly occupational limits for nuclear medicine technicians and radiochemists. However, for children and volunteers in research studies, this dose is still significant, particularly if repeated imaging is required [83]. CEST has the potential to allow more frequent imaging without a punitive radiation dose. As discussed above, CEST in its current form is a slow imaging technique with limited potential for whole body, and particularly 3D, image acquisition. In contrast, whole body FDG PET can be performed in 10–15 min on modern systems, with isotropic 3D image reconstruction and an inherently quantitative output. On the other hand, acquisition times are an issue when acquiring full Z-spectra but are not for dynamic scanning such as in [82]. Also, MRI historically has been able to solve such issues. For instance, data acquisition for the first fiber maps in animals [84] was a few hours, while it is now a few minutes for the clinic. Similar improvements could be expected for CEST.

Before directly comparing CEST techniques to PET, it is also important to carefully consider the pharmacological differences between the targets actually generating imaging contrast. Again, using Gluco-CEST and FDG PET as an example, it is clear that the Gluco-CEST signal is dependent on the concentration of unmetabolized glucose in each voxel. Thus, there is no distinction between glucose in blood, transiently present in a voxel, extracellular or intracellular glucose. Also, possible CEST effect of glucose metabolism intermediates can contribute to the measured signal and hamper glucose specificity. FDG PET detects the intracellular accumulation of FDG-6-P, the product of the enzyme hexokinase [85]. In the identification of tumors, this distinction is likely of low importance. However, it is not straightforward to study glucose transport or the actual rate of glucose metabolism with CEST. As a truly quantitative technique, kinetic models applied to dynamic PET data (with a knowledge of the system input, i.e., tracer in blood) can be used to quantify the activity of glucose transporters (e.g., GLUT1) transporting glucose from blood into cells, the activity of hexokinase, and overall glucose metabolism in μmol/min/100 g tissue [86]. Although this level of PET quantification is currently limited to research studies, efforts are being made to bring kinetic modeling into the clinic to improve the prognostic value of FDG PET still further.

The comparative merits and drawbacks of CEST versus PET are listed in Table 1. Table 2 provides a summary of the current status of CEST methods, as reported in the literature, in comparison with state-of-the-art clinical nuclear medicine. It is important, when considering this information, to keep in mind that PET is a mature (yet evolving) clinical modality, whereas CEST techniques are relatively new and undergoing a strong growth phase.

**Table 1:** Comparative summary of diaCEST vs PET

	CEST	PET
Resolution	On the order of 1–2 mm Depends on the hardware and pulse sequence, with no inherent limit. Generally a trade-off with acquisition time.	In the order of 4–5 mm Nonuniform across the field-of-view Intrinsically limited by positron range.
Sensitivity	Dependent on the targeted species as well as saturation scheme. Generally micro- to millimolar concentrations, although nanomolar concentrations have been reported in some studies.	Can detect picomolar concentrations of radiotracer.
Selectivity	Selective for exogenous CEST agents. Reduced selectivity for endogenous agents (several contributors, in addition to pH, temperature, buffer…)	Selectivity defined by radiotracer molecule.
Field-of-view	Inherently same as MR, in practice currently limited by the scan time. Typically single or a few 2D slices. 3D acquisition is also feasible.	Up to whole-body acquisition, in blocks defined by the axial coverage of the detector, typically 15-25 cm.
Scan length	Typically 1–2 min per sweep of the spectrum.	Typically 2 min per bed position for body imaging, 10 min for brain.
Risks	SAR caused by excessive RFs may lead to heating damage. This is prevented by built-in software safety measures.	A dose of ionizing radiation is delivered to the patient, both by the radiotracer itself and by the transmission scan used for attenuation correction purposes.
Pitfalls	At present, only a limited number of metabolites can be detected at 3 T. CEST acquisitions are severely limited by motion, especially in areas of B0 or B1 inhomogeneity. Reproducible CEST quantification is still under investigation.	Gamma attenuation information is required for quantitative reconstruction. This may be acquired by means of external gamma sources, or inferred from CT or MR images. A significant delay is often required between radiotracer injection and imaging, for redistribution and uptake purposes.

**Table 2:** Comparison of CEST with closest nuclear medicine alternatives

Target	Method	Contrast/mechanism	Preparation	Acquisition time^a^	Resolution^b^	Field strength	Status
Body tumors	Hydroxyl CEST	d-Glucose/glucose concentration	6 h fasting (for control)	N/A (preclinical)	N/A (preclinical)	9.4 T	Human studies
	FDG PET	[^18^F]Fluorodeoxyglucose/glucose metabolism ~350 MBq	4 h fasting 60 min uptake	~2 min/bed	~4 × 4 × 5 mm^3^	N/A	Clinical use
	Choline PET	[^18^F]Fluorocholine/cell membrane synthesis ~200 MBq	24 h diet 6 h fasting 2 min uptake	~2 min/bed 2 passes	~4 × 4 × 5 mm^3^	N/A	Clinical use
	PSMA PET	[^68^Ga]PSMA/prostate-specific membrane antigen ~150 MBq	60 min uptake	~4 min/bed	~4 × 4 × 5 mm^3^	N/A	Human studies
	DOTATATE PET DOTATOC PET DOTANOC PET	[^68^Ga]DOTA-conjugated peptides/somatostatin receptors ~150 MBq	60 min uptake	~2 min/bed	~4 × 4 × 5 mm^3^	N/A	Human studies
	FMISO PET	[^18^F]Fluoromisonidazole/macromolecules, hypoxia ~400 MBq	90 min uptake	~10 min/bed	~4 × 4 × 5 mm^3^	N/A	Human studies
Brain tumors	Amide CEST	None/protein concentration	Not needed	~40 s/slice	~2.2 × 2.2 × 4.4 mm^3^	3 T	Human studies
	Amine CEST	None/pH	Not needed	~1 min/slice	~2.3 × 2.3 × 6.0 mm^3^	3 T	Human studies
	FET PET	[^18^F]Fluoroethyltyrosine/amino acid ~130 MBq	4 h fasting 20 min uptake	~20 min dynamic	~4 × 4 × 5 mm^3^	N/A	Clinical use
	FLT PET	[^18^F]Fluorothymidine/DNA synthesis, proliferation ~250 MBq	45 min uptake	~60 min dynamic	~4 × 4 × 5 mm^3^	N/A	Human studies
	FDOPA PET	[^18^F]Fluoro-L-DOPA/dopamine receptors ~300 MBq	15 min uptake	~30 min dynamic	~4 × 4 × 5 mm^3^	N/A	Human studies
	FMISO PET	[^18^F]Fluoromisonidazole/macromolecules, hypoxia ~400 MBq	90 min uptake	~10 min	~4 × 4 × 5 mm^3^	N/A	Human studies
Stroke	Amide CEST	None/pH	Not needed	~13 s/slice	2.5 × 2.5 × 5.0 mm^3^	3 T	Human studies
	H_2_O/O_2_/CO PET	H_2_[15O]-O, [15O]-O_2_, C[15O]O/cerebral blood flow, oxygen consumption, blood volume (combined: oxygen extraction fraction) ~500 MBq	Not needed	~3 min dynamic	~4 × 4 × 5 mm^3^	N/A	Human studies
	NH_3_ PET	[^13^N]Ammonia/cerebral blood flow ~150 MBq	Not needed	~10 min dynamic	~4 × 4 × 5 mm^3^	N/A	Human studies
	FMISO PET	[^18^F]Fluoromisonidazole/macromolecules, hypoxia ~400 MBq	90 min uptake	~10 min	~4 × 4 × 5 mm^3^	N/A	Human studies
Neurological state	Amine CEST	None/glutamate concentration	Not needed	~10 min/slice	~1 × 1 × 5 mm^3^	7 T	Human studies
	Hydroxyl CEST	None/myo-inositol concentration Proposed marker of glial function/density	Not needed	N/A (preclinical)	N/A (preclinical)	9.4 T	Animal studies
	mGluR5 PET	[^11^C]-ABP, [^18^F]-PSS232/mGluR5 receptor density ~250 MBq	Not needed	~2 min/bed dynamic	~4 × 4 × 5 mm^3^	N/A	Human studies
	FDOPA PET	[^18^F]Fluoro-L-DOPA/dopamine receptors ~300 MBq	15 min uptake	~30 min dynamic	~4 × 4 × 5 mm^3^	N/A	Human studies
	Raclopride PET	[^11^C]Raclopride/dopamine receptors ~350 MBq	Not needed	~60 min dynamic	~4 × 4 × 5 mm^3^	N/A	Human studies
	FDG PET	[^18^F]Fluorodeoxyglucose/glucose metabolism ~350 MBq	4 h fasting 60 min uptake	~2 min/bed	~4 × 4 × 5 mm^3^	N/A	Clinical use
	Neurotransmitter transporter PET	Various/dopamine transporter, serotonin transporter	Various	Various	N/A	N/A	Animal studies
	SV2A PET	[^18^F]UCB-H, [^11^C]LEV/synaptic vesicle protein 2A (proposed marker of synaptic density)	Various	Various	N/A	N/A	Animal studies
Energetics	Amine CEST	None/creatine concentration	Not needed	~48 s/slice	~1 × 1 × 4 mm^3^	7 T	Human studies
	FDG PET	[^18^F]Fluorodeoxyglucose/glucose metabolism ~350 MBq	4 h fasting 60 min uptake	~2 min/bed	~4 × 4 × 5 mm^3^	N/A	Clinical use
Cartilage	Hydroxyl CEST	None/GAG concentration	Not needed	~30 s/slice	~0.7 × 0.7 × 3.0 mm^3^	7 T	Human studies
	FDG PET	[^18^F]Fluorodeoxyglucose/glucose metabolism ~350 MBq	4 h fasting 60 min uptake	~2 min/bed	~4 × 4 × 5 mm^3^	N/A	Clinical use

^a^PET acquisition times are provided per bed (i.e., anatomical station). The coverage of a PET bed varies with system geometry: PET/CT systems typically cover 70 × 70 × 15 cm, PET/MR systems 60 × 60 × 25 cm. Whole-body imaging (typically head-to-mid-thighs) requires between 6 and 8 beds

^b^The intrinsic resolution of PET systems decreases away from the center of the field-of-view (typically 1–2 mm). It is also dependent on the reconstruction algorithm. Standard measurements provided by manufacturers are performed using filtered backprojection, seldom used in clinical practice these days. The values provided here are approximations, representative of the average resolution that can be expected in state-of-the-art systems using iterative reconstruction methods. In the case of CEST, resolutions are not inherently limited but given by the scanner hardware, pulse sequence, and acquisition time. Typical values reported in recent literature are listed here as a reference

A separate note is deserved by the often overlooked potential synergies of these modalities. With the advance of simultaneous PET/MR data acquisition and multimodality data integration schemes, the prospect of CEST and PET imaging as complementary data streams is not unrealistic. In a first stage, hybrid systems provide an ideal platform for further development and cross-validation of both modalities. However, it is not hard to imagine certain indications where CEST and PET measurements could be used in combination, in order to maximize clinical information from a simultaneous PET/MR scan. For instance, large-FOV, high-specificity PET imaging could be leveraged to guide functionally complementary CEST (either single-voxel or imaging), not unlike some hybrid PET and MRS protocols currently under investigation.

## Conclusions

The appeal of CEST is obvious. Novel contrasts can be obtained using a variety of agents, endogenous, natural, or artificially synthesized compounds (including, in recent studies, drugs at pharmacological concentration). Compared to PET, CEST may eventually offer improved spatial resolution, lower cost, and reduced risk (no ionizing radiation). Compared to conventional MRI, CEST achieves unprecedented sensitivity at the molecular level.

The simultaneous operation of CEST and PET, as might be achieved with modern PET/MR systems, would be of tremendous interest, as the ability to obtain complementary information simultaneously may offer new insights into metabolic pathways and their pathology. Despite the limitations at currently approved clinical magnetic field strengths, the interest in CEST is rapidly growing.
